# Nuss Procedure for a Patient with Negative Haller Index

**DOI:** 10.1055/s-0038-1623537

**Published:** 2018-02-20

**Authors:** Mariela Dore, Paloma Triana Junco, Carlos De La Torre, Alejandra Vilanova-Sánchez, Monserrat Bret, Gaspar Gonzalez, Vanesa Nuñez Cerezo, Javier Jimenez Gomez, Jose Luis Encinas, Francisco Hernandez, Leopoldo Martínez Martínez, Manuel Lopez Santamaria

**Affiliations:** 1 Department of Pediatric Surgery, Hospital Universitario La Paz, Madrid, Madrid, Spain; 2 Department of Pediatric Radiology, Hospital Universitario La Paz, Madrid, Madrid, Spain; 3 Department of Pediatric Traumatology, Hospital Universitario La Paz, Madrid, Madrid, Spain

**Keywords:** MIRPE, Haller index, Nuss, pectus excavatum, children

## Abstract

**Introduction** Minimally invasive repair for pectus excavatum (MIRPE) is controversial in extremely severe cases of pectus excavatum (PE) and an open repair is usually favored. Our aim is to describe a case of a patient with an extremely severe PE that underwent a minimally invasive approach.

**Case report** An 8-year-old girl with severe sternum depression was assessed. She had a history of exercise intolerance, nocturnal dyspnea, fatigue, and shortness of breath. Chest computed tomography showed that sternum depression was posterior to the anterior vertebral column; therefore, Haller and correction index could not be measured. Spirometry indicated an obstructive ventilation pattern (forced expiratory volume in 1 second = 74.4%), and echocardiogram revealed a dilated inferior vena cava, mitral valve prolapse with normal ventricular function. After multidisciplinary committee evaluation, a MIRPE approach was performed.

All symptoms had disappeared at the 3-month postoperative follow-up; the desired sternum shape was achieved and normalization of cardiopulmonary function was observed. The Nuss bars were removed after a 2-year period. After 18-month follow-up, the patient can carry out normal exercise and is content with the cosmetic result.

**Conclusion** Nuss procedure is feasible in our 8-year-old patient. In this case, both the Haller and correction index were not useful to assess the severity of PE. Therefore, under these circumstances, other radiologic parameters have to be taken into consideration for patient evaluation.

## Introduction

Pectus excavatum (PE) is the most common anterior chest wall deformity. 1 2 3 The indication of surgical correction is based on the severity of the deformity, its progression, symptoms, functional testing, and the psychosocial effects on the patient. 4 5 Although the age of repair has been considered controversial, currently the indication of repair at any age is based primarily on the degree of the deformity and symptoms with evidence of cardiac and/or pulmonary compression. 6 The minimally invasive repair for pectus excavatum (MIRPE) was first described by Nuss in 1998. 4 7 8 Since then, its use has spread worldwide, modifications have been issued, and it is currently the procedure of choice in the correction of PE. However, some surgeons are still skeptic about its role in the surgical correction of extremely deep cases of PE.

The aim of this article is to describe the case of a patient with an extremely severe PE that underwent a minimally invasive approach, as well as the difficulties in assessing PE severity.

## Case Presentation

**Fig. 1:**
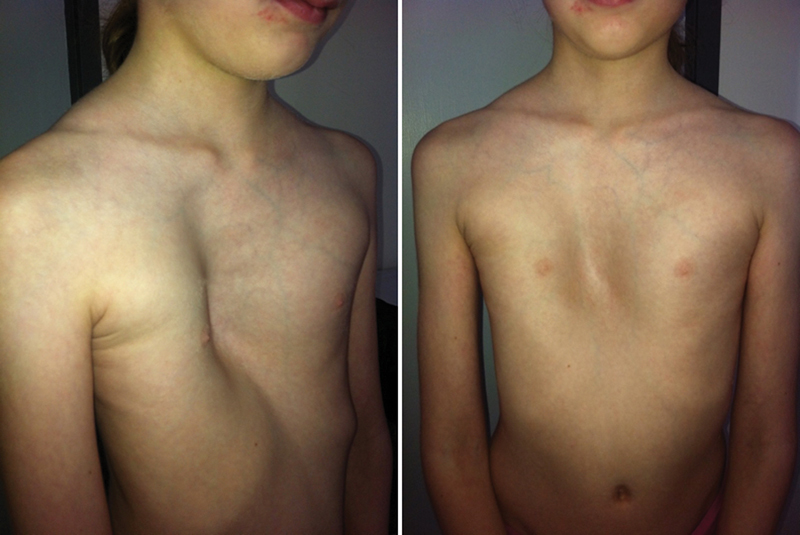
Patient prior to surgical intervention. Patient is an 8-year-old girl; image shows lateral and anteroposterior image at the time of the first outpatient clinic visit. An asymmetrical severe pectus excavatum is observed.

An 8-year-old girl with severe sternum depression and a history of exercise intolerance, fatigue, and shortness of breath on exertion was assessed. The patient also referred nocturnal dyspnea. Family history revealed an older sibling with asymptomatic severe PE that required surgical correction (Nuss procedure) at 7 years of age. Our patient's sternal depression was visible since birth and symptoms had started earlier in life (6–7 years of age), followed by rapid progression of symptoms which were more notable in the past 6 months. Genetic tests were performed to rule out Marfan syndrome as well as other abnormalities. The physical examination showed a prepubescent female with a height of 131 cm and weight of 22 kg. Her chest revealed a deep, asymmetric PE deformity ( Fig. 1 ).

**Fig. 2:**
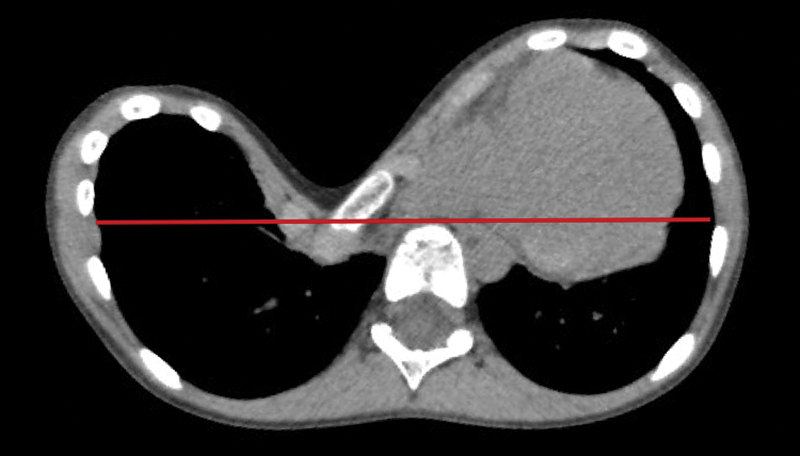
Patient's chest imaging prior to surgical correction. Chest computed tomography showing the sternum depression is below anterior vertebral column line (red line).

As part of the assessment protocol, a computed tomography (CT) scan was ordered (see Fig. 2 ). Chest CT evaluation showed that the sternum depression was posterior to the anterior vertebral column rendering the Haller and correction index, to the best of our knowledge unmeasurable. Other severity indexes were calculated: sternal rotation angle was 40° and the asymmetry index of 58%, indicating a marked left-sided asymmetry.

As part of the functional evaluation, a spirometry and echocardiogram were indicated. The preoperative spirometry indicated an obstructive ventilation pattern (forced expiratory volume in 1 second = 74.4%). The echocardiogram revealed a dilated inferior vena cava, mitral valve prolapse with normal ventricular function, all signs of ventricular compression.

After multidisciplinary committee evaluation, considering the severity of the symptoms and available resources, the indication of surgical repair was offered to the family. A MIRPE approach was designed. Right thoracoscopic visualization was achieved with a 0° and 10mm Storz endoscope placed at the fourth intercostal space. Although an angled scope offers a better view, it was not available at the time of the repair. This was followed by placement of a midsternum 7.5 mm cannulated bone screw (Laffitte). This would generate upward traction that would allow better visualization of the substernal dissection plane. After careful dissection, two 9-inch steel bars were slipped under the sternum through the fifth and seventh intercostal space following the Nuss procedure indications. Two steel bars and stabilizers on both sides of the bar were placed to fully correct the deformity and to prevent bar displacement.

**Fig. 3:**
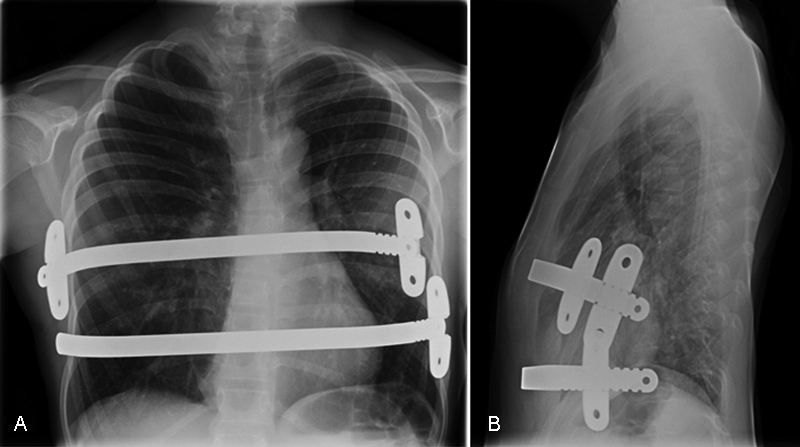
Postoperative results X-ray. ( **A** ) Anteroposterior (AP) chest X-ray after Nuss procedure. ( **B** ) Lateral chest X-ray after Nuss procedure. (c) AP chest X-ray after Nuss bar removal 2 years after placement.

**Fig. 4:**
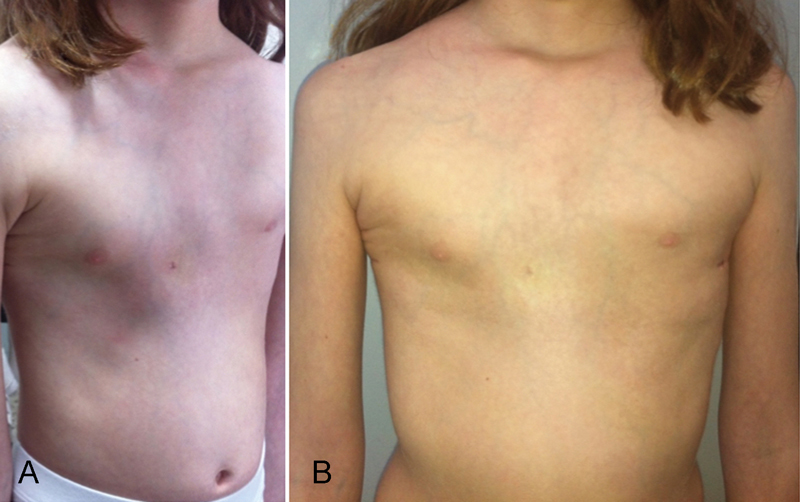
Postoperative image of patient. ( **A** ) Postoperative oblique/lateral view of patient's chest. ( **B** ) Postoperative anteroposterior view of patient's chest. A complete correction of the anterior chest wall deformity can be observed.

Postoperative care was uneventful allowing for a painless and quick recovery. At the 2 months' spirometry evaluation, an improvement in FEV1 and other parameters was observed (see Table 1 ), and cardiac compression signs disappeared on cardiac ultrasound. The patient referred that most symptoms had disappeared by the 3-month follow-up (nocturnal dyspnea and shortness of breath upon slight exertion), with great improvement in quality of life. Exercise was restricted during the initial 4 months, after which normal activity other than contact-sports were allowed. The desired sternum shape was achieved, with slight overcorrection noticed around 18 months postoperatively. The steel bars were removed after a 2-year period. At the 3-month follow-up, all of the patient's exercise intolerance had disappeared, and she was now more physically active. Postoperative bar removal imaging showed an acceptable correction of the deformity (see Figs. 3 and 4 ). During follow-up, the patient is content with the aesthetic result of the procedure; also the patient can carry out normal exercise.

**Table 1:** Spirometry parameters: preoperative versus postoperative

Observed/expected	Preoperative	Postoperative
**FEV1**	74.4%	81.4%
**FVC**	80.9%	84.6%
**FEV1/FVC ratio**	5.8%	2.3%
**MMEF 75/25**	48%	56.3%

Abbreviations: FEV1, forced expiratory volume in 1 second; FVC, forced vital capacity; MMEF 75/25, maximal midexpiratory flow.

## Discussion

Since 1998, when Nuss published the paper describing a minimally invasive repair, the open procedures have been replaced as the gold standard in the treatment of PE. 8 There have been modifications since its conception to improve reproducibility and prevent complications. The introduction of thoracoscopy, the development of bar stabilizers, as well as the addition of pericostal sutures have all contributed to minimize the number of complications associated with the substernal tunnel creation and displacement of the steel bars. 4 7

The indication of MIRPE over open repair in cases of severe PE is still cause for discussion. This is mainly due to the difficulty of the substernal dissection and steel bar placement due to the higher risk of cardiac injury in those patients with pectus eccentricity or sternal rotation that causes cardiac displacement. 9 The open repair, however, offers a higher complication rate. 10 This patient had one of the most severe PE repaired with a Nuss procedure in our institution, and as expected several concerns were raised. One of the concerns was the patient's age; however, Kelly et al had shared their experience in over 21 years of surgical correction of PE, in which age at the time of repair ranged from 1 to 31 years of age, and indication in these young patients was based on symptoms. 6 Park et al (How early) also offered results of repair in children over 3 years, with +600 cases of children under the age of five. Although follow-up is currently around 10 years, the recurrence rate remains low (0.4%) without statistical differences in morbidity or recurrence rate when compared with those patients who underwent repair at a later age. 11

There was also controversy as how to evaluate such a deformity. A debate was raised as to how to measure a Haller and correction index when the sternal depression is beyond the anterior vertebral column. Is it possible to measure the Haller or correction index in these patients? The correction index described by St Peter et al 12 used to obtain a quantitative measure of the severity of the deformity also requires the estimation of the minimal distance between the sternum depression and the anterior vertebral column. How do we adequately measure that centimeter that is beyond this line? Add a negative to the value?

Unfortunately, this case was evaluated prior to the introduction of cardiac magnetic resonance imaging (MRI) as part of our institution's assessment protocol of PE in 2015. 13 14 A chest CT with high radiation exposure has now been replaced by a cardiac MRI which offers inspiration/expiration imaging and shows the dynamics of the malformation, as well as a more integral assessment of its effect in cardiac function. 13 14 In this case, the calculation of other PE indexes was necessary to allow for proper measurement. The asymmetry index and angle rotation showed the magnitude of the deformity. 2 3 12 15 16

Other apprehensions were raised as the surgical plan was drawn. The main fear was the substantial risk of heart injury during substernal dissection and bar placement due to the severity of the malformation. To minimize this risk, available means were used to exert upward traction of the sternum that allowed better visualization. The vacuum bell, that is, now our current practice, was not available; hence, a bone screw was placed midsternum. 17 Nowadays, there are different tools that can be used for this purpose: vacuum bell, Uemura hooks, Dr. Park's Crane, Rultract retractor, among others. 5 18 de Campos and Tedde 18 describe several techniques that have allowed the upward traction of the sternum to make the repair safer. These procedures may be used not only in the case of severe PE but also in mild or moderate PE.

Another major concern was the risk of bar migration. Several studies have described this complication with rates ranging from 1% to 18%. 4 19 20 21 This was the main reason for placing two steel bars as well as stabilizers. Another technique described for this purpose is Park et al 22 bridge technique for PE bar fixation, in which one stabilizer is used to fixate both bars. Our choice was to place two steel bars and stabilizers on both sides of the bar to fully correct the deformity and to prevent bar displacement. However, our present practice is to place stabilizers on the left side of the bar (if the patient is right-handed) and to use pericostal sutures with Endoclose on the other side to achieve further stabilization.

Bar removal is usually performed after a 2- to 4-year period. 4 In this case, bar removal was scheduled after a 2-year period. This decision was made due to the slight overcorrection observed, and signs of bar impingement on the chest wall.

## Conclusion

PE is the most common anterior thoracic deformity. A MIRPE is widespread and has replaced open procedure for PE repair; however, extremely severe cases of PE continue to challenge surgeons. We believe that with current advances in imaging and surgical maneuvers, the repair of extremely severe PE with MIRPE is both feasible and safe. In the evaluation of this case, we are left with the uncertainty of how to measure the severity of PE since Haller and correction index in particularly deep PE cannot be calculated. In the meantime, we believe a negative Haller index and correction index describe the uniqueness and severity of this case.
